# Examination of Risk Factors and Expression Patterns of Atypical Femoral Fractures Using the Japanese Adverse Drug Event Report Database: A Retrospective Pharmacovigilance Study

**DOI:** 10.3390/ph16040626

**Published:** 2023-04-20

**Authors:** Shinya Toriumi, Ryuji Mimori, Haruhiko Sakamoto, Hitoshi Sueki, Munehiro Yamamoto, Yoshihiro Uesawa

**Affiliations:** 1 Department of Medical Molecular Informatics, Meiji Pharmaceutical University, Kiyose 204-8588, Japan; 2 Department of Pharmacy, National Hospital Organization Kanagawa Hospital, Hadano 257-8585, Japan; 3 Department of Orthopedic Surgery, National Hospital Organization Kanagawa Hospital, Hadano 257-8585, Japan

**Keywords:** atypical femoral fracture, bisphosphonates, denosumab, osteoporosis, cancer, Japanese Adverse Drug Event Report (JADER), multiple logistic regression analysis, Weibull distribution, epidemiological research, disproportionality analysis

## Abstract

Atypical femoral fracture (AFF) is a rare complication related to the use of bisphosphonates (BPs). Herein, we analyzed the risk factors and onset patterns of AFF using the Japanese Adverse Drug Event Report database and reported the findings. First, the independent risk factors for AFF were gender (female), high body mass index, and medical history of osteoporosis, arthritis, and systemic lupus erythematosus (SLE). Drug-related risk factors for AFF included BPs (i.e., alendronic acid, ibandronic acid, etidronic acid, zoledronic acid, minodronic acid, risedronic acid), denosumab, prednisolone, lansoprazole, rabeprazole, exemestane, letrozole, eldecalcitol, and menatetrenone. Therefore, it appears that AFF is influenced by a combination of patient backgrounds and drugs, and that the risk of developing AFF is particularly high in patients with fragile bones (e.g., osteoporosis, arthritis, and SLE). Second, in the analysis of AFF onset patterns, the onset of AFF from BPs and denosumab took a long time (>1 year) to develop. Analysis using a Weibull distribution showed wear-out failure-type AFF onset for BPs and denosumab, and both osteoporosis and cancer patients with long-term administration of these drugs showed a tendency to have an increased risk of onset. AFF developed earlier in osteoporosis patients with long-term administration of BPs and denosumab than in cancer patients.

## 1. Introduction

In 2005, Odvina et al. reported atypical femoral fracture (AFF) due to severely suppressed bone turnover resulting from the long-term use of bisphosphonates (BPs) [1]. AFF occurs in the subtrochanteric and diaphyseal areas and can be clearly distinguished from typical femoral fractures through characteristic imaging findings [2]. The clinical manifestations of AFF are stress or fragility fractures. AFFs are reported to be the most common drug-related fractures [3]. Although the mechanism of AFF remains unclear, the use of antiresorptive agents (such as BPs and denosumab) is a known risk factor [2,4]. While AFFs are rare adverse events associated with long-term administration of BPs and denosumab, they are difficult to assess accurately and they negatively affect the patient’s quality of life and prognosis [5]. Studying drug use in clinical settings is complex and differs from the adverse event profiles shown in well-designed clinical trials and epidemiologic studies. Adverse events that occur infrequently and only manifest after extended periods of drug administration, such as AFFs, are difficult to evaluate in pre-approval clinical studies.

BPs and denosumab are used for osteoporosis and bone lesions as they strongly suppress bone resorption via osteoclasts [6,7]. The main adverse events of these drugs include gastrointestinal symptoms, including stomach discomfort and constipation, and the rare adverse events include osteonecrosis of the jaw and AFF [8]. To reduce the risk of osteonecrosis of the jaw and AFF when using BPs and denosumab, assessment of the fracture risk after continuing treatment for 3–5 years is recommended and drug withdrawal needs to be considered [9,10].

In recent years, the large-scale spontaneous reporting system for recording adverse events in clinical settings has played a major role in epidemiological research centered on drug safety evaluation, because it now contains information collected over a long period of time. The Japanese Adverse Drug Event Report (JADER) database is a Japanese voluntary report database published by the Pharmaceuticals and Medical Devices Agency (PMDA) that contains approximately 1,290,000 records of adverse reactions of drugs used in Japan [11]. The adverse events recorded in the JADER database are presented in four tables and are publicly available. More than 90% of JADER reporters are healthcare professionals, and this database has more reports by healthcare professionals than in other spontaneous reports [12]. Since JADER contains drug administration dates and adverse event occurrence dates, adverse event-time analysis is facile [13]. By evaluating JADER, it may be possible to clarify the evaluation of drugs and correlate adverse event findings with the time of onset using clinical practice data.

The aim of this study is to clarify the risk factors and onset times of AFFs in clinical practice. Clarifying AFF onset is useful for the appropriate monitoring and management of BP and denosumab administration. Assisted by JADER—a spontaneous adverse reaction report database—we investigated the factors and expression patterns associated with AFF.

## 2. Results

### 2.1. Creation of Data Analysis Tables

JADER consists of a “DRUG” table hosting 4,154,715 records, a “REAC” table hosting 1,291,529 records, a “DEMO” table hosting 781,629 records, and a “HIST” table hosting 1,537,460 records. We combined the data from these four tables, removed 4809 ineligible records, and created a new table for data analysis. The data analysis table contained 2,034,718 records, of which 1879 reported AFFs.

### 2.2. Risk Factors for AFF

#### 2.2.1. Relationship between Patient Background and AFF

We compared the patient background characteristics associated with the AFF group (1879 reports) by simple regression analysis (Table 1). The mean gender, age, height, weight, body mass index (BMI), and medical history of the AFF group were female (1729 reports [94.4%]), 69.8 ± 13.8 years old, 151.6 ± 8.2 cm, 55.4 ± 14.5 kg, and 24.0 ± 5.6, and osteoporosis (757 reports [53.5%), cancer 368 reports [26.0%), arthritis 225 reports [15.3%), systemic lupus erythematosus (SLE) 136 reports [9.6%), and renal disorder (43 reports [3.0%)), respectively. In the non-AFF group (2,032,839 reports), the respective data were female (966,165 reports [48.9%]), 59.5 ± 21.5 years, 157.3 ± 18.4 cm, 54.6 ± 16.4 kg, and 21.9 ± 4.5, and osteoporosis (67,308 reports [4.4%], cancer 592.264 reports [39.0%], arthritis 120,158 reports [7.9%], SLE 91,005 reports [6.0%], and renal disorder 157,231 reports [10.3%]), espectively. Univariate regression analysis revealed significant differences in gender, age, height, BMI, osteoporosis, arthritis, and SLE.

#### 2.2.2. Relationship between Suspected Drugs and AFF

**Figure 1:**
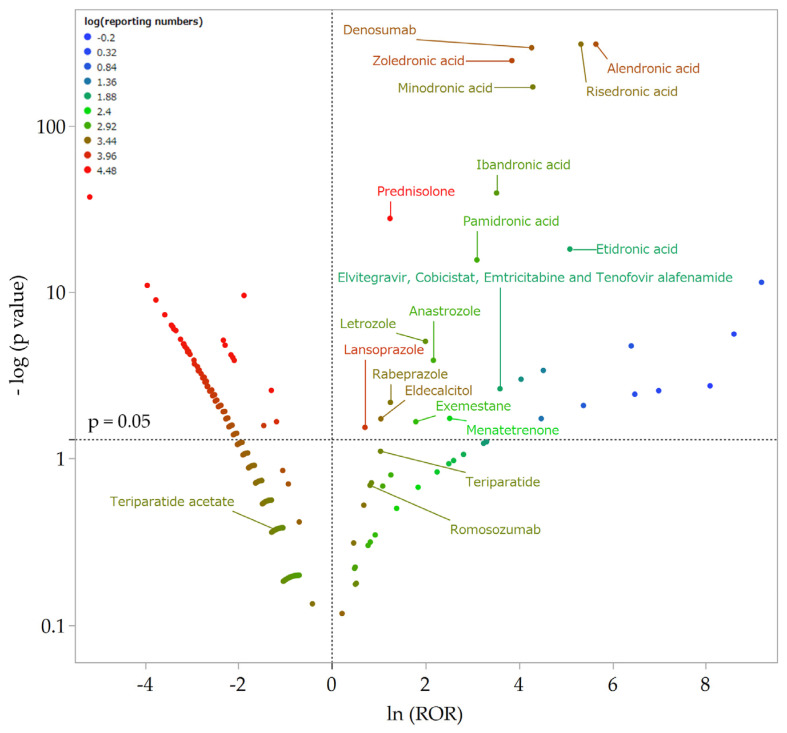
Volcano plot of drugs associated with AFF. The x-axis shows the natural logarithm of the odds ratios (ln (ROR)), while the y-axis shows the common logarithm of the inverse *p*-value (−log10 [*p*]) from Fisher’s exact test. The RORs were calculated using cross-tabulation. The dotted line on the y-axis represent *p* = 0.05. Plot colors represent the number of reports of adverse events. The red/green/blue points are common logarithms of the total reported numbers (range, −0.20 to 4.48). As the RORs become more positive, the tendency toward adverse events increases. Decreasing *p*-values indicate greater statistical significance. The upper-right portion of the plot identifies drugs that are more strongly associated with AFF.

A volcano plot was drawn to visually understand the relationships between the more than 4000 drugs reported in the JADER and AFF (Figure 1). The drugs plotted in the upper-right corner of the scatterplot are those likely to cause AFF (Table 2). In this study, 665 and 344 reports of alendronic acid and risedronic acid, respectively, were included as drugs with a high possibility of causing AFF. Teriparatide, teriparatide acetate, and romosozumab were not were notassociated with AFF.

#### 2.2.3. Multiple Logistic Regression Analysis

The results of the multiple logistic regression analysis are shown in Table 3. Independent risk factors for AFF were gender (female), high BMI, and medical history of osteoporosis, arthritis, and SLE. Administration of the following compounds was identified as independent risk factors: alendronic acid, ibandronic acid, etidronic acid, zoledronic acid, minodronic acid, risedronic acid, denosumab, rabeprazole, lansoprazole, prednisolone, exemestane, letrozole, menatetrenone, and eldecalcitol.

### 2.3. Onset Pattern Analysis Using Weibull Distribution

**Figure 2:**
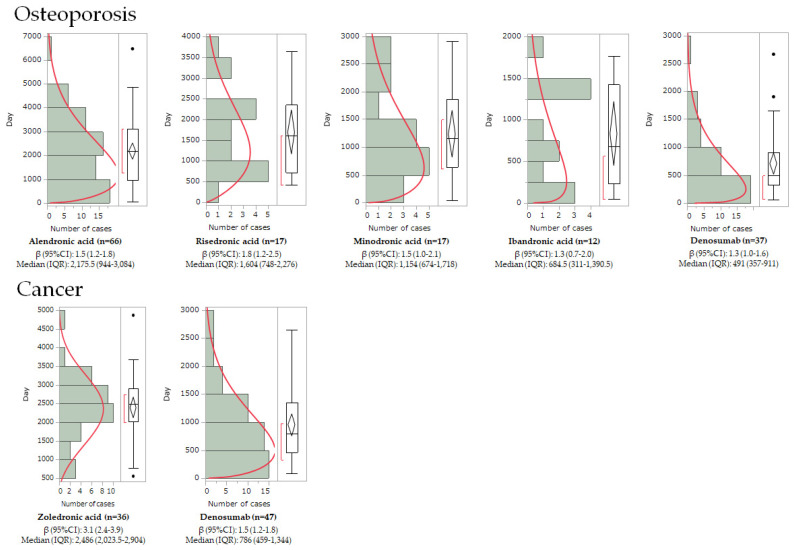
Time-to-onset analysis of BPs and denosumab in patients with osteoporosis and cancer. The y-axis indicates the days to onset, and the x-axis shows the number of reports (n). The left side of each graph is a histogram, while the right side is a box-and-whisker plot with outliers. The red line on the histogram was generated by fitting with a Weibull distribution.

Table 4 shows the median time to onset of AFF and the parameters of the Weibull distribution. For patients with osteoporosis, the median values for alendronic acid, risedronic acid, minodronic acid, ibandronic acid, and denosumab were 2176, 1604, 1122, 685, and 491 days, respectively. For cancer patients, the values for zoledronic acid and denosumab were 2486 days and 786 days, respectively. In patients with osteoporosis, the shape parameters (β) of alendronic acid, risedronic acid, minodronic acid, ibandronic acid, and denosumab were 1.5 (95% confidence interval (CI): 1.2–1.8), 1.8 (95% CI: 1.2–2.5), 1.5 (95% CI: 1.0–2.1), 1.3 (95% CI: 0.7–2.0), and 1.3 (95% CI: 1.0–1.6), respectively. In cancer patients, the values for zoledronic acid and denosumab were 3.1 (95% CI: 2.4–3.9) and 1.5 (95% CI: 1.2–1.8), respectively. Therefore, many BPs and denosumab had a shape parameter (β) of >1 and a lower bound of 95% CI > 1, indicating a wear-out pattern of onset with increasing risk over time. In addition, the curve fitted with a Weibull distribution (red curve) peaked earlier in osteoporotic patients than in cancer patients (Figure 2).

## 3. Discussion

### 3.1. Risk Factors for AFF

The comprehensive analysis of patient backgrounds and over 4000 drugs identified specific patient factors and 13 drugs as risk factors for AFF. Therefore, the first finding suggests that AFF may be affected by a combination of patient backgrounds and drugs that affect bone health.

In the multiple logistic regression analysis of this study, the patient factors associated with AFF were females with high BMI, and co-presentation with osteoporosis, arthritis, and SLE (Table 3). In a Japanese survey, 23 of 24 AFF patients were women [14]. Furthermore, high BMI has been reported to increase the risk of AFF [15,16]. This study further corroborates these references, as 92.0% of the AFF cases in this study were female, and high BMI was associated with an increased risk of developing AFF. Regarding medical history, osteoporosis is a disease in which bone strength decreases due to decreased bone density and deterioration of bone quality [17]. Both SLE and arthritis are inflammatory diseases. Corticosteroid administration is considered to be the main cause of bone fragility in SLE [18] and arthritis [19], but the disease itself may also be involved. In SLE, various immune cells and synovial epithelial cells have been reported to produce inflammatory cytokines such as IL-1 [20], IL-6 [21], and TNF-α [22]. Increased levels of IL-4, IL-6, TNF-α, IL-10, and IL-17 have been reported in rheumatoid arthritis [23]. These inflammatory cytokines can activate osteoclasts and enhance bone resorption [24]. Therefore, it is conceivable that patients with these diseases would have bone fragility. Overall reduction in bone strength, such as osteoporosis, was found to be a possible trigger for AFF. Contrastingly, cancer was not identified as a risk factor for AFF in this study.

In this study, six BPs, (zoledronic acid, alendronic acid, minodronic acid, risedronic acid, ibandronic acid, and etidronic acid) and denosumab were associated with AFF (Table 3). BPs have a strong affinity for bone hydroxyapatite, inhibit osteoclast activity, and decrease bone resorption. BPs are typically prescribed for the treatment of osteoporosis and malignant tumors [25]. A meta-analysis found that the use of BPs increased the relative risk of AFF by 28-fold [26]. In this study, based on the JADER database, many records indicated drugs used for osteoporosis patients, such as alendronate with 665 records, risedronic acid with 344 records, and minodronic acid with 120 records (Table 2). However, there were also 201 records of zoledronic acid used for cancer treatment (Table 2). Regardless of the reason for their prescription, the use of BPs requires attention and continuous vigilance toward the development of AFF. Denosumab was also an independent risk factor for AFF in this study (Table 3). Denosumab is an anti-receptor activator for nuclear factor-κB ligand antibody (anti-RANKL antibody) that, like BPs, has an inhibitory effect on osteoclasts and is used for malignant tumors and osteoporosis [27,28]. The half-life of denosumab is approximately 26 days and shorter than that of BPs [29]; however, denosumab has been similarly reported to cause AFF [30,31]. Paradoxically, bone resorption inhibitors—such as BP and denosumab—cause AFF and should be treated with caution. In contrast, the new osteoporosis drugs teriparatide and romosozumab were not associated with AFF in this study (Table 2). These drugs have been proposed as alternatives to BPs and denosumab [32], and this is supported by the present study.

This study suggested that prednisolone—a corticosteroid—may be associated with AFF (Table 3). Long-term administration of corticosteroids is known to cause frequent pathological fractures. Daily oral steroid treatment with ≥5 mg of prednisolone equivalents is known to reduce bone mineral density [33]. The onset mechanism of steroid-induced osteoporosis includes a direct effect on the bone metabolism system—mainly suppressing osteogenic cells such as osteoblasts—and an indirect effect via the endocrine system. Corticosteroids also act to promote osteocyte apoptosis [34,35], inhibit osteoclast apoptosis [36], and suppress sex hormone (estrogen, testosterone, etc.) secretion by suppressing the production of gonadotropin-releasing hormone (GnRH) [37]. Corticosteroids have also been reported to inhibit calcium absorption in humans and promote urinary calcium excretion. In this study, it was found that prednisolone may affect bone fragility, because low bone density was associated with AFF.

In this study, the proton-pump inhibitors (PPIs) rabeprazole and lansoprazole were associated with AFF (Table 3). PPIs are acid secretion inhibitors used for peptic ulcers and gastroesophageal reflux disease. Long-term administration or high-dose prescriptions of PPIs, has been reported to increase the risk of bone fractures [38,39]. This suggests that PPIs reduce intestinal calcium absorption by inhibiting gastric acid secretion [40]. Furthermore, in animal studies, PPIs were found to decrease serum calcium levels, decrease osteocalcin levels, and growth plate thickness [41], inhibit osteoclast H+/K+ ATPase pumps [42], and reduce osteoclast viability and bone resorption markers [43]. In this study, PPIs were associated with AFF and with common fracturesby inhibiting bone resorption.

The aromatase inhibitors exemestane and letrozole, which are therapeutic agents for breast cancer, were associated with AFF (Table 3). Exemestane is a nonsteroidal-type aromatase inhibitor, while letrozole is a steroidal-type aromatase inhibitor. Both of these aromatase inhibitors are prescribed in first-line postoperative hormone therapy for postmenopausal breast cancer. Aromatase inhibitors have the effect of lowering blood estrogen levels. Blood estradiol levels are closely related to bone metabolism in postmenopausal women, and there is a correlation between low blood estradiol levels and bone mineral density [44,45]. Therefore, it is conceivable that aromatase inhibitors reduce bone density and weaken bones, leading to AFF.

### 3.2. Patterns for AFF Onset Timelines 

The second finding in this study was that AFF induced by BP and denosumab occurred after long-term administration, and the shape parameter (β) of the Weibull distribution showed an onset pattern of wear-out failure. Additionally, when these drugs were prescribed for osteoporosis, AFF developed earlier than when these drugs were prescribed for cancers.

In this study, many of the BPs resulted in AFF after several years of administration (Table 4) and showed similar onset patterns (Figure 2). The risk of AFF increased with increasing duration of BP use, with a hazard ratio of 8.86 (95% CI: 2.79–28.20) for 3 to less than 5 years and 43.51 (13.70–138.15) over 8 years [46]. This study supports known findings in both cancer and osteoporosis patients. Denosumab also showed an onset pattern similar to that of BPs, and an increased risk of AFF was observed with long-term use (Table 4). Therefore, administration of BPs and denosumab requires continuous monitoring for adverse events, including AFF, during long-term use rather than immediately after administration.

We also found that patients with osteoporosis may develop AFF earlier than those with cancer. The median values for AFF onset in osteoporosis patients treated with BPs (685–2176 days) and denosumab (491 days) were earlier than for cancer patients treated with zoledronic acid (2486 days) and denosumab (786 days) (Table 4). BPs and denosumab in patients with osteoporosis also peaked earlier than those in patients with malignant tumors (Figure 2). In the search for risk factors in the previous section, osteoporosis was found to be a risk factor, but malignancy was not (Table 2). Considering these facts, it is conceivable that osteoporosis, which causes bone fragility, contributes to the onset of AFF. In general, the dosages of BPs and denosumab in cancer patients for bone lesions were higher than for osteoporosis; however, the effect of dosage on the onset time was limited. Correlations between purpose of administration and dosage to onset time are a subject for future investigation.

### 3.3. Limitations

This study has three limitations [47,48]: First, as the adverse event database is based on spontaneous reports, the reported cases are limited to those recognized as adverse event. Therefore, since the total number of patients using each drug was unknown in this study, it was not possible to truly evaluate adverse event. Second, the JADER data report contained blank cells and typographical errors. Therefore, some of the data values for adverse event and drug names were manually corrected to the best of our ability, and patient backgrounds were evaluated using BMI as the most consistently presented data parameter. Third, it is difficult to identify the cause of adverse event when multiple drugs are administered. While the adverse event of “fatality” in the JADER data could be verified by PMDA, other reported adverse event were subjectively based on the reporter’s judgment. Thus, because of some limitations in the use of the JADER database for analysis, we often validate the results of the adverse event database by conducting follow-up studies, such as epidemiological ones. Nevertheless, JADER is the largest database for spontaneously reported adverse reactions in Japan, and adverse drug reaction information obtained from JADER is considered to reflect not only specific pharmacological and pharmacokinetic characteristics, but also prescription and usage conditions. Therefore, JADER is an excellent tool for broadly identifying the adverse event of drugs that can be used in many diverse fields of research.

## 4. Materials and Methods

### 4.1. Preparation of JADER and Data Tables for Analysis

This study analyzed data registered in the JADER database from 1 April 2004 to 31 August 2022, were used for the analysis in this study [11]. JADER consists of four tables: DRUG (drug name, causal relationship, etc.), REAC (adverse events, outcome, etc.), DEMO (patient demographic information, such as gender, age, weight, etc.), and HIST (medical history, primary disease, etc.). Based on their involvement in adverse events, the drugs registered in the DRUG table are assigned to three categories: suspect drugs, concomitant drugs, and interactions. In this study, only the suspect drugs’ data were extracted from the DRUG table. Adverse events in the REAC table and underlying diseases in the HIST table are based on the ICH International Medical Dictionary for Regulatory Activities/Japanese version 25.0 (MedDRA/J ver.25.0) [49]. Adverse events in the REAC table and underlying diseases in the HIST table can be grouped by the standardized MedDRA query (SMQ)—the standard search formula that comprehensively captures the related MedDRA preferred term and system organ classes. In this study, primary diseases in the HIST table were grouped into osteoporosis, cancer, SLE, arthritis, and renal disorder using SMQ (Table 5). In this study, the SMQ grouping for primary disease used a “broad: wide area” term to detect all cases that may indicate the condition. The preferred terms associated with grouped primary diseases are listed in Supplementary Table S1. Duplicate cases in the DRUG and REAC tables were eliminated using the method reported by Hirooka et al. [50,51]. The four tables (DRUG table, REAC table, DEMO table, and HIST table) were combined using identification numbers. Furthermore, in order to exclude nonconforming reports in this study, BMI was calculated from the combined data table, and reports with less than 10 (3427 reports) or greater than 100 (1382 reports) were excluded. In this study, we analyzed the entries reported as “atypical femoral fractures” by JADER.

### 4.2. Risk Factors for AFFs

. In this study, AFF was used as the outcome variable, and patient background and drugs as predictor variables Patient background, age, sex, weight, height, and medical history (osteoporosis, cancer, arthritis, SLE, renal disorder) were examined. Regarding patient background, age, height, and weight, and BMIdata were treated as continuous variables, and *p*-values were calculated USING the Wilcoxon rank-sum test. Weight in the 60 kg range was converted to 65, and ages below 10 years were converted to 5. Sex and medical history were treated as binary variables. For sex, the *p*-value was calculated using Fisher’s exact two-sided test. Past medical history was determined via Fisher’s exact right-sided test. For each patient factor, only data without missing values were analyzed [52].

**Figure 3:**
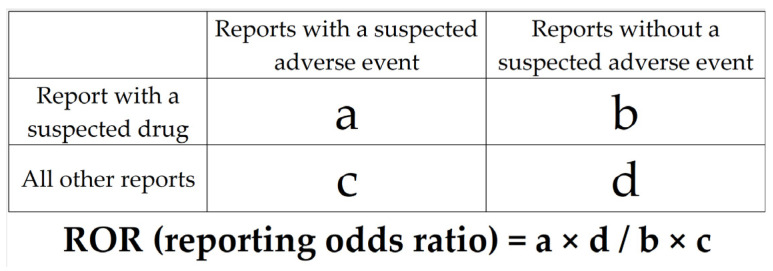
Cross-tabulation and formula used to calculate the ROR for an adverse event. The cross-tabulation is structured with reports for the suspected drug, all other reports, reports with an adverse event, and reports without an adverse event (a–d indicate the number of reports).

Drugs were treated as binary variable, and all available drugs were analyzed exhaustively. A 2 × 2 contingency table for each drug and AFF was generated and assessed using reported odds ratios (RORs) and Fisher’s exact *p*-values (Figure 3). The 2 × 2 contingency table was corrected by adding 0.5 to all cells, because the estimation is unstable if each cell is 0 or smaller (Haldane–Anscombe 1/2 correction) [53,54]. In this study, drugs with RORs of 1 or greater and Fisher’s exact *p*-values of 0.05 or less were considered to be potentially associated with AFF. For the purpose of visual interpretation of the relationships between drugs and AFF, a scatterplot (volcano plot) consisting of RORs and *p*-values for all drugs was generated. The scatterplot used in this study corresponds to the volcano plot, which is frequently used to understand gene expression trends in the bioinformatics field [55,56]. This plot was produced by transforming RORs to natural logarithms (ln (ROR)) on the y-axis and transforming Fisher’s exact test *p*-values to common logarithms [−log (*p*-value)] on the x-axis. The number of reported cases for each drug was indicated by color, and drugs with the highest reported number of cases were indicated with red dots. Therefore, drugs plotted in the upper-right quadrant and marked with a red dot had a high possibility of causing AFF.

Multiple logistic regression analysis was performed using patient characteristics and medications that were found to be associated with AFF from the previous section. In the analysis, AFF was used as outcomevariable, and the patient background and drug factors as predictor variables [57,58]. For each drug, a dummy variable was generated and coded +1 and −1: for example, etidronic acid was defined as 1 and other drugs as −1. Multiple logistic regression analysis was performed using 643,125 reports with missing values removed from the analytical data table.

### 4.3. Onset Pattern Analysis of AFF by Weibull Distribution

Expression pattern analysis of AFF was analyzed for BPs and denosumab administered for cancer and osteoporosis. The JADER data included the start date of drug administration and the date of adverse event onset. The adverse event occurrence period was calculated by “adverse event occurrence date − administration start date + 1”, and the characteristics of AFF were analyzed using the histogram and Weibull distributions [59,60]. The Weibull distribution is the distribution of failure rates with respect to time. The scale parameter (α) indicates the spread of the distribution, and the shape parameter (β) indicates the occurrence pattern of failures. The shape parameter (β) indicates the change in failure at a point in time. An early failure type is classified with a decreasing rate of occurrence over time (β < 1), a random failure type is classified with a constant rate of occurrence (β = 1), and a wear-out failure type is classified with an increasing rate of occurrence over time (β > 1). The Weibull distribution employed in this study is typically used in industrial quality control. In this study, failure was interpreted as the occurrence of an adverse pharmaceutical event.

### 4.4. Statistical Analysis

All analyses were performed using JMP Pro 16.2.0 (SAS Institute Inc., Cary, NC, USA), and *p* < 0.05 was considered significant.

## 5. Conclusions

In this study, JADER—a spontaneous adverse reaction reporting database—was mined to determine factors and expression patterns associated with AFF, resulting in two main findings: First, the drugs that increase the risk of AFF included BPs and denosumab (associated with osteoclast suppression), PPIs, estrogen inhibitors, and corticosteroids (associated with bone fragility). The patient risk factors for AFF included being female, high BMI, osteoporosis, arthritis, and SLE. Therefore, AFF may have multiple risk factors that act in combination. Second, according to the Weibull distribution analysis, AFF induced by BPs and denosumab was of a wear-out failure type, and long-term administration of these drugs contributed to increased risk of AFF onset. Patients diagnosed with osteoporosis developed AFF earlier than patients diagnosed with cancer; therefore, patients with fragile bones—such as those suffering from osteoporosis—continuously taking BPs and denosumab should be monitored carefully for signs of AFF.

## Figures and Tables

**Table 1:** Comparison of patient backgrounds and AFF (n = 2,034,718).

Patient Backgrounds	AFF (n = 1879)	Non-AFF (n = 2,032,839)	*p*-Value
Sex (male/female) ^#^	102/1729 (1831)	1,003,206/966,165 (1,977,662)	<0.001 ^###^
Age *	69.8 ± 13.7 (1638)	59. 5± 21.5 (1,895,047)	<0.001 ***
Height (cm) *	55.4 ± 14.5 (395)	54.6 ± 16.4 (919,036)	0.07
Weight (kg) *	151.6 ± 8.2 (378)	157.3 ± 18.4 (786,612)	<0.001 ***
BMI *	24.0 ± 5.6 (378)	21.9 ± 4.5 (763,182)	<0.001 ***
Cancer ^†^	368 (1414)	592,264 (1,520,573)	1.0
Osteoporosis ^†^	757 (1414)	67,308 (1,520,573)	<0.001 ^†††^
Arthritis ^†^	225 (1414)	120,158 (1,520,573)	<0.001 ^†††^
SLE ^†^	136 (1414)	91,005 (1,520,573)	<0.001 ^†††^
Renal disorder ^†^	43 (1414)	157,231 (1,520,573)	1.0

**AFF, atypical femoral fracture; BMI, body mass index; SLE, systemic lupus erythematosus.** Some values were missing for each variable; analyses were performed using data after eliminating these records. The numbers in parentheses are the numbers of cases used in the analyses. ^#^ Fisher’s exact test (two-tailed test); * Wilcoxon signed-rank test; ^†^ Fisher’s exact test (right one-tailed test); ^###^, ***, ^†††^: *p* < 0.001.

**Table 2:** Comparison of drugs associated with AFF (n = 2,034,718).

Drug	Drug Class	AEF (1879)	Non-AFF (2,037,648)	Reporting Ratio	ROR	95% Confidence Interval	*p*-Value
Alendronic acid	BP	665	3937	14.50%	283.02	256.20–312.65	<0.001 **
Risedronic acid	BP	344	2222	13.40%	205.47	181.50–232.60	<0.001 **
Zoledronic acid	BP	201	5197	3.70%	46.94	40.46–54.47	<0.001 **
Minodronic acid	BP	120	1898	5.90%	73.44	60.73–88.80	<0.001 **
Ibandronic acid	BP	35	1158	2.90%	33.83	24.15–47.39	<0.001 **
Pamidronic acid	BP	16	811	1.90%	22.22	13.62–36.26	<0.001 **
Etidronic acid	BP	10	70	12.50%	162.33	84.75–310.92	<0.001 **
Denosumab	Anti-RANKL antibody	210	3580	5.50%	71.63	61.83–82.98	<0.001 **
Prednisolone	Corticosteroid	119	39,152	0.30%	3.46	2.88–4.17	<0.001 **
Lansoprazole	PPI	13	7240	0.20%	2.03	1.19–3.47	0.029 *
Rabeprazole	PPI	7	2333	0.30%	3.49	1.70–7.16	0.007 *
Letrozole	Aromatase inhibitor	9	1399	0.60%	7.39	3.90–14.01	<0.001 **
Anastrozole	Aromatase inhibitor	6	806	0.70%	8.76	4.04–18.98	<0.001 **
Exemestane	Aromatase inhibitor	3	631	0.50%	6.02	2.10–17.22	0.022 *
Eldecalcitol	Vitamin D	7	2860	0.20%	2.85	1.39–5.84	0.019 *
Menatetrenone	Vitamin K	2	218	0.90%	12.42	3.57–43.23	0.018 *
Teriparatide	Parathyroid hormone	4	1727	0.23%	2.83	1.11–7.14	0.078
Teriparatide acetate	Parathyroid hormone	0	1810	0.00%	0.30	0.02–4.79	0.423
Romosozumab	Sclerostin inhibition	3	1678	0.18%	2.26	0.79–6.46	0.204
Elvitegravir, Cobicistat, emtricitabine, and tenofovir alafenamide fumarate	HIV drugs	2	74	2.60%	36.42	10.32–128.52	0.002 *

**AFF, atypical femoral fracture; ROR, reporting odds ratio; BP, bisphosphonate; Anti-RANKL, anti-receptor activator of nuclear factor-κ B ligand; PPI, proton pump inhibitor; HIV, human immunodeficiency virus.** Reporting ratio is the proportion of AFF in the total number of adverse events for each drug. Analyses were conducted after eliminating records with missing data. *: *p* < 0.05, **: *p* < 0.001.

**Table 3:** Multiple logistic regression analysis (n = 643,125).

Risk Factor	Drug Class	Odds Ratio	95% Confidence Interval	*p*-Value
Etidronic acid	BP	1150.66	318.65–4155.08	<0.001 **
Alendronic acid	BP	502.28	324.05–778.54	<0.001 **
Minodronic acid	BP	390.66	225.92–675.53	<0.001 **
Risedronic acid	BP	343.26	202.95–580.56	<0.001 **
Zoledronic acid	BP	300.67	184.95–488.80	<0.001 **
Ibandronic acid	BP	132.99	53.51–330.5	<0.001 **
Denosumab	Anti-RANKL antibody	705.40	464.27–1071.76	<0.001 **
Prednisolone	Corticosteroid	26.35	15.28–45.45	<0.001 **
Rabeprazole	PPI	39.12	9.38–163.09	0.001 *
Lansoprazole	PPI	18.28	5.61–59.53	0.001 *
Exemestane	Aromatase inhibitor	58.39	7.93–429.77	0.013 *
Letrozole	Aromatase inhibitor	24.86	3.38–182.84	0.034 *
Eldecalcitol	Vitamin D	16.25	3.83–69.03	0.007 *
Menatetrenone	Vitamin K	289.92	67.35–1247.98	<0.001 **
Female	N/A	4.02	2.81–5.74	<0.001 **
Osteoporosis	N/A	2.53	1.91–3.37	<0.001 **
SLE	N/A	1.91	1.27–2.89	0.004 *
Arthritis	N/A	1.36	1.01–1.82	0.037 *
Age (unit)	N/A	0.99	0.99–1	0.137
Age (range)	N/A	0.52	0.24–1.15	0.137
BMI (unit)	N/A	1.11	1.09–1.13	<0.001 **
BMI (range)	N/A	3047.24	775.66–11,971.25	<0.001 **

**BP, bisphosphonate; Anti-RANKL, anti-receptor activator of nuclear factor-κ B ligand; PPI, proton-pump inhibitor; SLE, systemic lupus erythematosus; BMI, body mass index; N/A, not applicable.** Analyses were conductedafter eliminating records with missing data. *: *p* < 0.05, **: *p* < 0.001.

**Table 4:** Median and Weibull distribution of AFF.

Drug	Drug Class	n	Median	Interquartile Range		Scale Parameter		Shape Parameter		
				25%	75%	α	95% CI	β	95% CI	Pattern
**Osteoporosis**										
Alendronic acid	BP	66	2176	944	3084	2398.7	2017–2835.3	1.5	1.2–1.8	wear-out failure
Risedronic acid	BP	17	1604	748	2276	1919.1	1408.3–2564	1.8	1.2–2.5	wear-out failure
Minodronic acid	BP	17	1122	604	1718	1360.8	937.6–1936.5	1.5	1.0–2.1	wear-out failure
Ibandronic acid	BP	12	685	311	1390	889.8	519.4–1473.8	1.3	0.7–2.0	wear-out failure
Denosumab	Anti-RANKL antibody	37	491	357	911	769.5	585–1000	1.3	1.0–1.6	wear-out failure
**Cancer**										
Zoledronic acid	BP	36	2486	2023	2904	2668.3	2377.3–2982.1	3.1	2.4–3.9	wear-out failure
Denosumab	Anti-RANKL antibody	47	786	459	1344	1053.1	851.3–1291.2	1.5	1.2–1.8	wear-out failure

**BP, bisphosphonate; Anti-RANKL, anti-receptor activator of nuclear factor-κ B ligand.** Drugs with a calculated β of 1 or more and a 95% CI lower limit value not including 1 were presumed to be wear-out failure-types, in which AFF tends to occur late.

**Table 5:** Definition of medical history.

Osteoporosis		Cancer			
Code	SMQ	Code	SMQ	Code	SMQ
20000178	Osteoporosis/osteopenia	20000092	Malignant-disorder-related state	20000203	Prostate tumor unidentified in detail
Arthritis		20000094	Tumor marker	20000204	Malignant skin tumor
Code	SMQ	20000110	Neoplasm of the oropharynx	20000205	Skin tumor unidentified in detail
20000216	Arthritis	20000194	Malignant tumor	20000206	Malignant uterus/salpingioma
Systemic lupus erythematosus		20000195	Tumor unidentified in detail	20000207	Uterus/salpingioma unidentified in detail
Code	SMQ	20000196	Malignant biliary tract neoplasm	20000208	Malignant hepatophyma
20000045	Systemic lupus erythematosus	20000197	Biliary tract neoplasm unknown in detail	20000209	Hepatophyma unidentified in detail
Renal disorder		20000198	Malignant breast tumor	20000215	Malignant lymphoma
Code	SMQ	20000199	Breast tumor unknown in detail		
20000003	Acute renal failure	20000200	Malignant ovarian tumor		
20000181	Renal vessel disorder	20000201	Ovarian tumor unidentified in detail		
20000213	Chronic kidney disease	20000202	Malignant prostate tumor		

**Medical history was examined for osteoporosis, cancer, arthritis, systemic lupus erythematosus, and renal disorder.** Each medical history was grouped using standard MedDRA queries (SMQs). Renal disorder was grouped using 3 SMQs, and cancer was grouped using 20 SMQs.

## Data Availability

Data is contained within the article. Data from the JADER database were downloaded from the website of the Pharmaceuticals and Medical Devices Agency (https://www.pmda.go.jp/) on August 2022.

## Supplementary Materials

The following supporting information can be downloaded at: https://www.mdpi.com/article/10.3390/ph16040626/s1, Table S1: Preferred terms related to grouped medical history.
